# An Integrated Gene Regulatory Network Controls Stem Cell Proliferation in Teeth 

**DOI:** 10.1371/journal.pbio.0050159

**Published:** 2007-06-12

**Authors:** Xiu-Ping Wang, Marika Suomalainen, Szabolcs Felszeghy, Laura C Zelarayan, Maria T Alonso, Maksim V Plikus, Richard L Maas, Cheng-Ming Chuong, Thomas Schimmang, Irma Thesleff

**Affiliations:** 1 Developmental Biology Programme, Institute of Biotechnology, Viikki Biocenter, University of Helsinki, Finland; 2 Division of Genetics, Department of Medicine, Brigham and Women's Hospital and Harvard Medical School, Boston, Massachusetts, United States of America; 3 Center for Molecular Neurobiology Hamburg, University of Hamburg, Hamburg, Germany; 4 Institute for Biology and Molecular Genetics, Superior Research Council and University of Valladolid, Valladolid, Spain; 5 Department of Pathology, Keck School of Medicine, University of Southern California, Los Angeles, California, United States of America; Duke University Medical Center, United States of America

## Abstract

Epithelial stem cells reside in specific niches that regulate their self-renewal and differentiation, and are responsible for the continuous regeneration of tissues such as hair, skin, and gut. Although the regenerative potential of mammalian teeth is limited, mouse incisors grow continuously throughout life and contain stem cells at their proximal ends in the cervical loops. In the labial cervical loop, the epithelial stem cells proliferate and migrate along the labial surface, differentiating into enamel-forming ameloblasts. In contrast, the lingual cervical loop contains fewer proliferating stem cells, and the lingual incisor surface lacks ameloblasts and enamel. Here we have used a combination of mouse mutant analyses, organ culture experiments, and expression studies to identify the key signaling molecules that regulate stem cell proliferation in the rodent incisor stem cell niche, and to elucidate their role in the generation of the intrinsic asymmetry of the incisors. We show that epithelial stem cell proliferation in the cervical loops is controlled by an integrated gene regulatory network consisting of Activin, bone morphogenetic protein (BMP), fibroblast growth factor (FGF), and Follistatin within the incisor stem cell niche. Mesenchymal FGF3 stimulates epithelial stem cell proliferation, and BMP4 represses *Fgf3* expression. In turn, Activin, which is strongly expressed in labial mesenchyme, inhibits the repressive effect of BMP4 and restricts *Fgf3* expression to labial dental mesenchyme, resulting in increased stem cell proliferation and a large, labial stem cell niche. Follistatin limits the number of lingual stem cells, further contributing to the characteristic asymmetry of mouse incisors, and on the basis of our findings, we suggest a model in which Follistatin antagonizes the activity of Activin. These results show how the spatially restricted and balanced effects of specific components of a signaling network can regulate stem cell proliferation in the niche and account for asymmetric organogenesis. Subtle variations in this or related regulatory networks may explain the different regenerative capacities of various organs and animal species.

## Author Summary

Stem cells reside in specific niches that regulate their self-renewal and differentiation, and are responsible for the continuous regeneration of tissues. Although the regenerative potential of mammalian teeth is limited, mouse incisors grow continuously throughout life and contain stem cells at their proximal ends in the so-called cervical loops. We have used a combination of mouse mutant analyses, organ culture experiments, and gene expression studies to identify the key signaling molecules that regulate epithelial stem cell proliferation in the cervical loop stem cell niche. We show that signals from the adjacent mesenchymal tissue regulate epithelial stem cells and form a complex regulatory network with epithelial signals. Stem cell proliferation is stimulated by fibroblast growth factor 3 (FGF3), and bone morphogenetic protein 4 (BMP4) represses *Fgf3* expression. In turn, Activin inhibits the repressive effect of BMP4 and Follistatin antagonizes the activity of Activin. We also show that spatial differences in the levels of Activin and Follistatin expression contribute to the characteristic asymmetry of rodent incisors, which are covered by enamel only on their labial (front) side. We suggest that subtle variations in this or related regulatory networks may explain the different regenerative capacities and asymmetric development of various organs and animal species.

## Abstract 3

A network comprising Activin, BMP, FGF, and Follistatin regulate incisor stem cell proliferation in the niche and account for asymmetric organogenesis.

## Introduction

**Figure 1:**
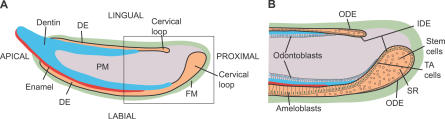
Schematics of the Continuously Growing Mouse Incisor (A) Basic overall organization of the incisor tooth. Growth occurs from proximal to apical (incisal) end. At the proximal end lie the lingual and labial cervical loops, each containing epithelial stem cells. (B) Enlargement of boxed region in (A) showing the individual cell types that comprise the proximal end. Stem cells reside within stellate reticulum, core of the cervical loop. See text for details. Color coding is as follows: enamel (red), dentin (blue), epithelium (orange), and follicular mesenchyme (green). DE, dental epithelium, FM, follicle mesenchyme, IDE, inner dental epithelium, ODE, outer dental epithelium, PM, papilla mesenchyme, SR, stellate reticulum, TA, transit amplifying.

Stem cells reside in specific niches that regulate their self-renewal and differentiation. In general, these niches consist of various types of neighboring differentiated cells, which provide a milieu of extracellular matrix and signaling molecules that regulate the unique behavior of stem cells. Epithelial stem cells have been identified in several adult tissues that undergo continuous turnover, such as hair, skin, feather, and gut [1–6]. Although mammals have lost the capacity for recurrent tooth renewal, some mammals, such as rodents, have incisor teeth that grow continuously. On the basis of cell cycle kinetics, DiI tracing, and the location of cells that retain bromodeoxyuridine (BrdU) label long term, it is thought that the epithelial stem cells of rodent incisors reside in the stellate reticulum core of the cervical loops (Figure 1A and 1B; [1,7]). An interesting feature of the mouse incisor is that the cervical loop on the labial side is much thicker than that on the lingual side. It contains abundant stellate reticulum cells in its core, whereas the lingual cervical loop is very thin and only contains a few stellate reticulum cells. However, the molecular mechanisms underlying the asymmetric growth and size of these cervical loops are unknown.

Most dental epithelial stem cells give rise to enamel-secreting ameloblasts. The enamel layer covers the dentin layer produced by mesenchymal odontoblasts. Besides the asymmetric growth and morphology of the cervical loops, enamel also exhibits asymmetric distribution in the mouse incisors. Enamel-producing ameloblasts differentiate only along the labial aspect of the mouse incisor, so enamel covers only the labial surface of the tooth, whereas the lingual surface is enamel-free and covered only by dentin (Figure 1A). This, together with the continuous growth and wear of the mouse incisor, maintains its characteristic sharpness, which is crucial to its function for gnawing. We showed previously that ameloblast differentiation on the labial side of the incisor is induced by bone morphogenetic protein 4 (BMP4) from mesenchymal odontoblasts, and that Follistatin inhibits BMP function in lingual-side dental epithelium, preventing enamel formation there [8]. Consistent with this, ameloblasts differentiate on both sides of incisors in *Follistatin^−/−^* mice, whereas overexpression of *Follistatin* in the dental epithelium (*Keratin 14 [K14]-Follistatin* mice) inhibits ameloblast differentiation and enamel formation. Hence, the asymmetric expression of *Follistatin* in the dental epithelium contributes to the asymmetry of enamel formation in the mouse incisors [8]. However, although the basis for the selective differentiation of ameloblasts on the labial incisor surface is partly understood, the mechanisms that underlie the regulation of the stem cells that give rise to ameloblast progenitors is unknown.

The dental mesenchyme has important functions in influencing the epithelial stem cell niche. Fibroblast growth factor (FGF) signals from the mesenchyme have been shown to regulate Notch signaling in the epithelium [1], and FGF10 has been identified as a necessary signal for epithelial stem cell maintenance, based on the hypoplastic morphology of the cervical loop in *Fgf10^−/−^* mice [9]. Although FGF10 and presumably Notch1 are important in stem cell maintenance, they are expressed in similar patterns on both the labial and lingual sides of the mouse incisor [1,10]. So far, the earliest gene reported to be expressed asymmetrically in the developing mouse incisor is *Fgf3*. From embryonic day 16 (E16) onwards, *Fgf3* expression is restricted to a small area of dental papilla mesenchyme on the labial side directly underneath the thick labial cervical loop [9].

The present study started from the observation that incisor growth was markedly slowed in *K14-Follistatin* mice, and that the cervical loops were severely hypoplastic, whereas in *Follistatin^−/−^* mutants, the lingual cervical loop was enlarged. This suggested the hypothesis that Follistatin might in some way inhibit the maintenance or proliferation of dental epithelial stem cells. We show that a complex, highly integrated, and spatially regulated signaling network underlies the effect. *Fgf3* expression in the mesenchyme underlies the cervical loops and correlates with their size, and FGF3 cooperates with FGF10 as a mesenchymal signal that stimulates the proliferation of dental epithelial stem cells and transit amplifying (TA) cells. BMP4 represses *Fgf3* expression, whereas Activin, which is preferentially expressed in labial mesenchyme, inhibits the repressive effect of BMP4 and restricts *Fgf3* expression to the labial dental mesenchyme, resulting in the large, labial-side stem cell niche. Moreover, unlike its function in ameloblast differentiation, Follistatin may antagonize the function of Activin, rather than BMP in the cervical loop area, and thereby limit the proliferation of lingual stem cells. This results in a small, lingual stem cell niche and, collectively, accounts for the asymmetry of the incisor. Thus, the number of dental epithelial stem cells and their spatial disposition are regulated by a complex signaling network involving Activin, BMP, FGF, and Follistatin that mediates the communication between mesenchymal and epithelial cells in the stem cell niche. These results show how the spatial regulation of signals can account for asymmetric organogenesis

## Results/Discussion

**Figure 2:**
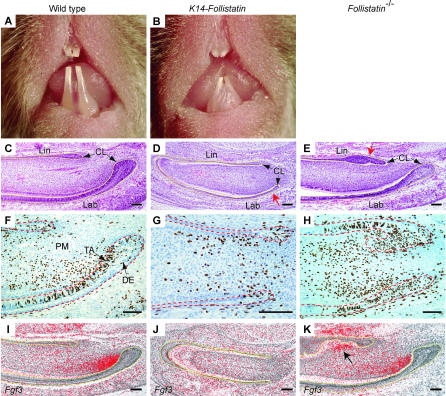
Follistatin Inhibits the Proliferation of Epithelial Stem Cells and TA Cells and the Expression of *Fgf3* in the Mouse Incisor (A and B) At P17, the growth of *K14-Follistatin* mouse incisors is retarded relative to wild type. (C) At the newborn stage, wild-type incisors exhibit a large, labial (Lab) cervical loop (CL; arrow) and a much smaller lingual (Lin) cervical loop. (D) In newborn *K14-Follistatin* mice, the labial cervical loop is severely hypoplastic (red arrow). (E) In newborn *Follistatin^−/−^* incisors, the lingual cervical loop is hyperplastic (red arrow). (F–H) BrdU incorporation experiments at E18 reveal a marked reduction in cell proliferation in *K14-Follistatin* incisors, especially in the TA cell region of the labial dental epithelium (DE). In *Follistatin^−/−^* incisors, there is an increase in cell proliferation in the lingual dental epithelium. Dashed red lines indicate the epithelium. (I–K) In situ hybridization analysis of *Fgf3* expression (red color represents signal). (I) In newborn wild-type mice, *Fgf3* is expressed asymmetrically in dental papilla mesenchyme adjacent to the labial cervical loop. (J) *Fgf3* expression is markedly down-regulated in P1 *K14-Follistatin* incisors. (K) *Follistatin^−/−^* incisors exhibit ectopic *Fgf3* (arrow) in lingual dental mesenchyme adjacent to proliferating epithelial cells. Yellow lines indicate the epithelium (C–E, I–K). Scale bar represents 100 μm (C–K).

We observed that incisor growth was markedly slowed in *K14-Follistatin* transgenic mice that overexpress the transforming growth factor β (TGF-β) antagonist Follistatin in the dental epithelium (Figures 2A, 2B, and S1). Histological analysis showed that in contrast to wild type, the labial cervical loop of *K14-Follistatin* newborns was severely hypoplastic and resembled the lingual cervical loop in size (Figure 2C and 2D). Conversely, in *Follistatin^−/−^* embryos, which die at birth [8,11], the lingual cervical loops frequently showed marked overgrowth and resembled the labial cervical loop in size (Figure 2E). These observations suggested that Follistatin influences epithelial stem cell proliferation in the incisor tooth germ.

To test this hypothesis, we performed BrdU incorporation experiments to assay cell proliferation. In wild-type mice, proliferative activity was most obvious in the labial inner dental epithelium, representing actively dividing TA cells that undergo further division and gradually become pre-ameloblasts and ameloblasts. BrdU-labeled cells were less apparent in the central core of the cervical loop where putative slow-cycling stem cells are located, and were very sparse in lingual cervical loop epithelium (Figure 2F). In contrast, in *K14-Follistatin* mice, the number of stellate reticulum cells, as well as proliferating epithelial cells, were markedly decreased in the labial cervical loop (Figures 2G and S1). Hence, *Follistatin* overexpression strongly inhibits dental epithelial cell proliferation and results in a compromised stem cell niche. Conversely, *Follistatin^−/−^* incisor germs contained numerous BrdU-positive cells in both labial and lingual cervical loops (Figures 2F, 2H, and S1). This switch from lingual to labial cervical loop morphology in *Follistatin^−/−^* embryos demonstrates that Follistatin functions in the generation of incisor asymmetry through inhibition of epithelial stem cell and TA cell proliferation.

The maintenance and proliferation of stem cells in the cervical loop epithelium depends upon FGFs that are expressed in the subjacent dental mesenchyme [1,9]. *Fgf3* expression partly overlaps that of *Fgf10,* but has a more restricted pattern in that it underlies only labial-side TA cells. In vitro bead implantation assays showed that, similar to FGF10 [1], FGF3 stimulated epithelial cell proliferation in E15 incisors (Figure S1). In *K14-Follistatin* incisors, *Fgf3* expression was completely down-regulated (Figure 2I and 2J). Moreover, in *Follistatin^−/−^* mice, *Fgf3* was ectopically expressed in lingual mesenchyme adjacent to the TA cells of the enlarged cervical loop (Figure 2K). In contrast, *Fgf10* expression appeared unchanged from wild type (unpublished data). These results indicate that Follistatin prevents the expression of *Fgf3* in the lingual dental mesenchyme, whereas forced expression of *Follistatin* leads to down-regulation of *Fgf3* in the labial epithelium.

**Figure 3:**
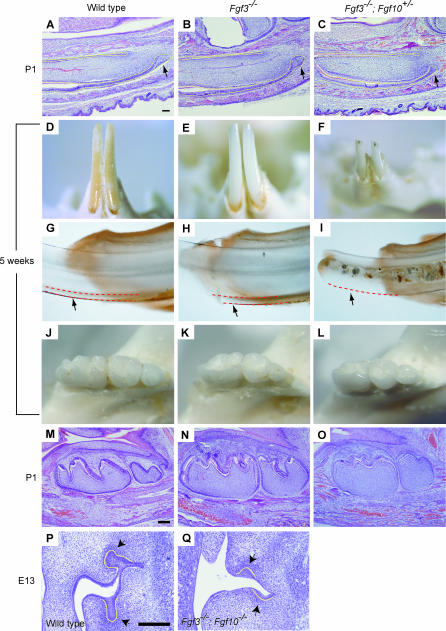
*Fgf* Genetic Interactions in Incisor and Molar Development (A–I) Lower incisor phenotypes in wild type and in *Fgf3^−/−^* and *Fgf3^−/−^; Fgf10^+/−^* mutants. (A–C) Histology at P1 shows wild-type and *Fgf3^−/−^* incisor development are indistinguishable, whereas *Fgf3^−/−^; Fgf10^+/−^* incisors are smaller and exhibit hypoplastic labial cervical loops (arrows). (D–F) Gross photographs of 5-wk-old incisors. Note the absence of pigmentation in *Fgf3* mutants, and the thin, broken incisors in a *Fgf3^−/−^; Fgf10^+/−^* mutant. (G–I) Ground sections of incisors in D-F, respectively, showing equivalent enamel layer thickness (dashed red lines) in wild-type and *Fgf3^−/−^* mutants, and enamel layer absence in a *Fgf3^−/−^; Fgf10^+/−^* mutant (arrows). (J–L) Five-week-old *Fgf3^−/−^* and *Fgf3^−/−^; Fgf10^+/−^* lower molars are smaller than normal, and enamel is prematurely worn on the occlusal surface. (M–O) At P1, folding of *Fgf3^−/−^* molar epithelium is aberrant and shallower than normal; this phenotype is more severe in *Fgf3^−/−^; Fgf10^+/−^* molars. (P and Q) Histological sections of E13 molar region in wild-type and *Fgf3^−/−^; Fgf10^−/−^* double mutants show that the development of both maxillary and mandibular molars is arrested prior to bud stage in the mutant. One out of 90 double-mutant embryos survived until E13. Scale bar represents 100 μm (A–C and M–Q).

Next we analyzed incisor development in *Fgf3^−/−^* and *Fgf3^−/−^; Fgf10^+/−^* compound mutants. In *Fgf3^−/−^* mutants at postnatal day 1 (P1), labial cervical loop morphology and ameloblast differentiation appeared normal (Figure 3A and 3B). *Fgf10^−/−^* mice die at birth with hypoplastic cervical loops, whereas *Fgf10^+/−^* mice exhibit no abnormalities in incisors or molars [9]. In *Fgf3^−/−^; Fgf10^+/−^* compound mutants at P1, however, the labial cervical loops were smaller than wild type (Figure 3C). Moreover, at 5 wk, both *Fgf3^−/−^* and *Fgf3^−/−^; Fgf10^+/−^* lower incisors were white and lacked characteristic yellow-brown pigment, and the compound mutant incisors were thin and frequently broken (Figure 3D–3F). Ground sections of *Fgf3^−/−^* incisors showed that an enamel layer was present (Figure 3G and 3H), but a defect in enamel structure was indicated by the premature wear of molar teeth (Figure 3K). Ground sections of *Fgf3^−/−^; Fgf10^+/−^* incisors showed that the enamel layer was either very thin or missing (Figure 3I).

In addition, the molars of *Fgf3^−/−^* and *Fgf3^−/−^; Fgf10^+/−^* mice were smaller than wild-type teeth, and at P1, the folding of *Fgf3^−/−^* molar epithelium was aberrant, the cusps were shallow, and this phenotype became more severe in *Fgf3^−/−^; Fgf10^+/−^* molars (Figure 3K, 3L, 3N, and 3O). These data indicate that FGF3 and FGF10 function cooperatively during tooth development. This was further supported by the observation that in an *Fgf3^−/−^; Fgf10^−/−^* double null mutant embryo, molar development was arrested prior to the bud stage (Figure 3Q), whereas in the single knockouts, the molars, albeit smaller than wild type, underwent complete development (Figure 3K; [9]).

It appears that although FGF3 and FGF10 are not required for the early differentiation of ameloblasts during embryonic crown morphogenesis [9], they regulate the maintenance and proliferation of epithelial stem cells in the cervical loops later in the erupted incisors. Thus, during incisor development, *Fgf3* and *Fgf10* genetically interact to maintain the epithelial stem cell pool that provides a continuous supply of ameloblast progenitors. Moreover, although FGF3 is not necessary for early cervical loop morphogenesis, the asymmetric expression of FGF3 appears to be sufficient to generate the asymmetric labial and lingual stem cell niches in mouse incisors. Several lines of evidence support this view. First, FGF3 protein induces intense cell proliferation in incisor epithelium (Figure S1). Second, *Fgf3* is expressed asymmetrically in incisor dental mesenchyme, in a spatial manner that correlates with differences in cervical loop sizes. Last, ectopic *Fgf3* expression was detected in the lingual dental mesenchyme underlying the enlarged cervical loop in *Follistatin^−/−^* incisors. These data suggest a model whereby the lingual cervical loop receives only an FGF10 signal, which maintains the basal epithelial stem cell proliferation that would be required for the continuous incisor growth. In contrast, the labial aspect receives both FGF3 and FGF10 signals, resulting in a larger stem cell niche with an increased number of proliferating stem cells. Because the cervical loop defects in *K14-Follistatin* mice are more severe than those in *Fgf3^−/−^* mice, other molecules besides FGF3 may also be affected by Follistatin overexpression.

**Figure 4:**
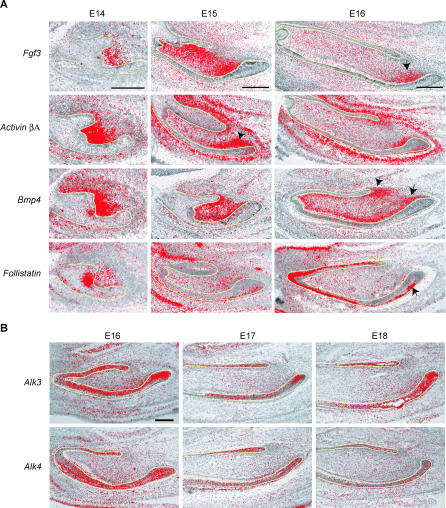
Signaling Molecule and Receptor Gene Expression in Incisor Dental Mesenchyme In situ hybridization for the indicated genes in incisor tooth germs from E14 to E18. (A) Note the symmetric mesenchymal expression of *Bmp4* in the cervical loop regions and the onset of asymmetric mesenchymal expression of *Activin βA* and *Fgf3* at E15 and E16, respectively (arrows). *Follistatin* is expressed throughout the thin layer of lingual dental epithelium as well as in the labial outer dental epithelium, but is down-regulated in the inner dental epithelium labially (arrow). (B) The BMP and Activin receptors Alk3 and Alk4, respectively, are expressed in the epithelium on both labial and lingual sides between E16 and E18. Scale bar represents 200 μm.

To explore the mechanism by which Follistatin, an extracellular antagonist of Activin and BMP [12], inhibited *Fgf3* expression, we first compared the expression of *Fgf3, Activin βA, Bmp2, Bmp4, Bmp7,* and *Follistatin* during incisor development. In E14 dental mesenchyme, striking co-expression of *Fgf3, Activin βA,* and *Bmp4* was observed; *Follistatin* was also expressed in the mesenchyme, but at higher levels in adjacent dental epithelium (Figure 4A). At E15, mesenchymal *Fgf3* and *Bmp4* expression persisted whereas *Activin βA* became restricted to the labial mesenchyme directly underlying the cervical loop epithelium; much weaker expression was observed around the lingual cervical loop (Figure 4A). At E16, *Fgf3* expression also became asymmetric, being undetectable lingually and overlapping with *Activin βA* labially; *Bmp4* was intensely expressed around both lingual and labial cervical loops, as well as in mesenchymal preodontoblasts on both sides. *Bmp2* and *Bmp7* were expressed symmetrically in dental papilla mesenchyme at all the stages examined (Figure S2). *Follistatin* was expressed widely in E15 dental epithelium. However, at E16, *Follistatin* was down-regulated in the labial inner dental epithelium, but persisted in the outer dental epithelium. Hence, there was an obvious gap between these *Follistatin*-expressing cells and the dental papilla mesenchyme in the cervical loop area. On the lingual side, the thin layers of dental epithelium continued to express *Follistatin* intensely (Figure 4A). Activin receptor-like kinase 3 (Alk3), the BMP receptor type 1A, and Alk4, the Activin receptor type 1B, were expressed intensely in the incisor epithelium (Figure 4B).

**Figure 5:**
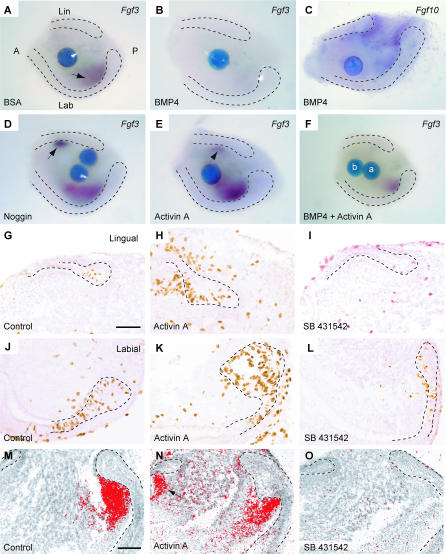
BMP4 Inhibits *Fgf3* Expression in Dental Mesenchyme and Activin Counteracts This Effect and Stimulates Cell Proliferation (A–F) E16 incisor explants containing the cervical loop region were cultured in vitro for 24 h with beads containing the indicated factors, and gene expression was analyzed by whole-mount in situ hybridization. (A) In BSA bead controls, *Fgf3* is expressed only in labial (Lab) dental mesenchyme underlying the epithelium (arrow, 46/46). (B) BMP4 beads down-regulate *Fgf3* expression (39/47). (C) *Fgf10* expression is unaffected by BMP4 beads (14/14). (D) Noggin beads induce ectopic *Fgf3* expression in lingual (Lin) mesenchyme directly adjacent to the inner dental epithelium (arrow, 8/18). (E) Activin A beads also induce ectopic *Fgf3* expression in lingual mesenchyme (arrow, 21/35). (F) When both BMP4 and Activin A beads (a) are inserted, the repression of *Fgf3* expression by BMP4 (b) is abolished (4/7). A, anterior, P, posterior. (G–O) When P1 incisor explants are cultured in the presence of Activin A for 4 d, cell proliferation is stimulated as assayed by BrdU incorporation, and both lingual (G and H) and labial (J and K) cervical loops are increased in size (21/25). Ectopic *Fgf3* is induced in the lingual dental mesenchyme directly underneath the enlarged cervical loop (arrow, [M] and [N]). When the inhibitor of Activin receptor-like kinase (ALK) receptors (SB431542) is added to the culture medium of incisor explants, the labial cervical loop is thinner, containing fewer stellate reticulum cells, and epithelial cell proliferation is reduced in both labial and lingual cervical loops (11/12) (I and L). In these samples, *Fgf3* is down-regulated in the dental mesenchyme (M and O). Dashed lines indicate the epithelium. Scale bar represents 100 μm (G–O).

To determine the inter-relationships between the different components of this gene regulatory network, we used organ cultures of dissected E16 incisors. Because we had shown previously that Follistatin antagonizes the inductive effect of BMP in differentiating ameloblasts [8], we first inserted beads soaked in recombinant BMP4 into the cervical loop region of incisor explants and examined the expression of *Fgf3* and *Fgf10*. Unexpectedly, after 1 d in culture, *Fgf3* expression was markedly down-regulated by BMP4 in the dental mesenchyme, whereas BMP4 had no effect on *Fgf10* expression (Figure 5A–5C). We next inserted beads soaked in the BMP inhibitor Noggin into the incisor cervical loop region. Ectopic *Fgf3* expression was strongly induced in the lingual dental mesenchyme directly underlying the dental epithelium (Figure 5D, arrow), confirming that BMPs inhibit *Fgf3* expression in incisor dental mesenchyme.

Consistent with this conclusion, *K14-Noggin* transgenic mice that overexpress Noggin in dental epithelium exhibit markedly overgrown incisors with increased epithelial proliferation in both the labial and lingual cervical loops [13]. Similar to *Follistatin^−/−^* mice and Noggin bead experiments, we detected ectopic *Fgf3* expression in the mesenchyme underlying the lingual cervical loops in *K14-Noggin* incisors (Figure S3). Collectively, these results demonstrate that BMPs repress *Fgf3* expression in dental mesenchyme in vivo, thereby negatively regulating epithelial stem cell proliferation in the mouse incisor.

The observation that Follistatin and BMPs have similar effects on the cervical loop, i.e., they both inhibit *Fgf3* expression and growth of the epithelium, indicates that Follistatin is unlikely to act as a BMP antagonist in the cervical loop. We therefore chose to investigate the activity of Activin A, another target of Follistatin, in this system. Remarkably, similar to Noggin beads, Activin A beads also induced ectopic *Fgf3* expression in lingual incisor mesenchyme directly underlying the dental epithelium (Figure 5E, arrow). Thus, Activin A and BMP4 have opposite effects on *Fgf3* expression. When Activin A and BMP4 beads were placed together in incisor explants, the inhibition of *Fgf3* by BMP4 was abrogated (Figure 5F). Next, we studied the effect of Activin on the proliferation of the cervical loop epithelium in cultured explants. When present for 1 or 2 d in incisor explant culture medium, Activin A did not significantly affect cervical loop growth (unpublished data). However, after 4 d, it had induced marked overgrowth in both labial and lingual cervical loops, with extra budding and increased cell proliferation (Figures 5G, 5H, 5J, and 5K). Consistent with the bead experiments, ectopic *Fgf3* was also induced in the lingual dental mesenchyme directly underneath the enlarged cervical loop (Figure 5M and 5N). Because the Activin null mutant mice lack incisors and die shortly after birth [14], we used the selective inhibitor of ALK receptors (SB431542) to prevent endogenous Activin/TGF-β signaling in the cultured incisor explants [15]. The addition of SB431542 in culture medium had no detectable affect after 1 or 2 d of culture (unpublished data), but after 4 d, the labial cervical loop was thinner than normal, with a reduced number of stellate reticulum cells, and epithelial cell proliferation was decreased in both labial and lingual cervical loops (Figure 5I and 5L). Also *Fgf3* expression was down-regulated in the dental mesenchyme (Figure 5M and 5O). These findings supported the role of Activin as a positive regulator of epithelial stem cell proliferation.

**Figure 6:**
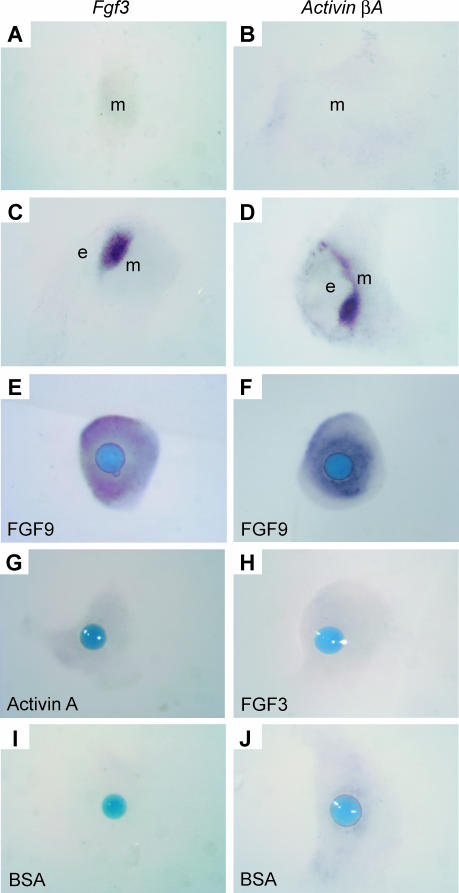
Dental Epithelium Maintains *Activin βA* and *Fgf3* Expression in Mesenchyme, and This Effect is Mimicked by FGF9 (A and B) When incisor mesenchyme (m) is isolated from dental epithelium (e) and cultured in vitro for 1 d, both *Fgf3* (E14: 0/4; E15: 0/15) and *Activin βA* (E14: 0/3) expression are down-regulated to undetectable levels. (C and D) When isolated dental epithelium and mesenchyme are recombined, strong *Fgf3* (E14: 10/11; E15: 13/15) and *Activin βA* (E14: 9/9) expression is induced in the mesenchyme at the epithelial-mesenchymal boundary. (E and F) FGF9 beads induce strong expression of *Fgf3* (E14: 7/7; E15: 3/3) and *Activin βA* (E14: 6/6; E15: 2/2) in incisor mesenchyme around the bead. (G) Activin A beads do not induce *Fgf3* expression in isolated dental mesenchyme (E15: 0/13). (H) FGF3 beads do not induce *Activin βA* in isolated dental mesenchyme (E15: 0/14). (I and J) No induction of *Fgf3* (E14: 0/4; E15: 0/6) or *Activin βA* (E14: 0/3; E15: 0/6) occurs around BSA control beads.

Several lines of evidence support the idea that the effects of Activin and BMP on *Fgf3* expression are indirect and that they are likely mediated via the epithelium. First, Activin beads did not induce *Fgf3* expression in dental mesenchyme when the dental epithelium was removed, indicating either an indirect effect or a co-requirement for additional factor(s) (Figure 6G). Second, the effects of Activin and the Activin inhibitor on epithelial proliferation was delayed, being detectable after 4 d in culture (Figure 5G, 5H, 5J, and 5K), but not after 1 d (Figure S1, and unpublished data). Third, the ectopic *Fgf3* expression in the Activin bead experiments was localized directly underneath the dental epithelium, not around the Noggin or Activin beads (Figure 5D and 5E). Fourth, the BMP and Activin receptors, Alk3 and Alk4, respectively, were intensely expressed in the dental epithelium. These results suggest that the regulation of *Fgf3* expression in the dental mesenchyme by Activin and BMP is indirect and that dental epithelium is an indispensable part of this regulatory network. Thus, Activin acts as a positive regulator, whereas BMP4 is a negative regulator of *Fgf3* expression, and hence of epithelial stem cell proliferation in mouse incisors, and the balance between these two mesenchymally expressed TGF-β superfamily members dictates the rate of epithelial stem cell proliferation. The finding that the preferential expression of *Activin* in labial mesenchyme precedes that of *Fgf3* by a day (Figure 4A) further supports the conclusion that *Activin* acts upstream of *Fgf3*.

Lastly, we addressed the regulation of *Fgf3* and *Activin* expression in incisor mesenchyme. Beads soaked in Activin A did not induce *Fgf3* expression and neither did FGF3 induce *Activin* in dental mesenchyme (Figure 6G and 6H). Both dental epithelium and beads soaked in FGF9 efficiently induced *Fgf3* and *Activin* mesenchymal expression in explants (Figure 6C–6F). However, *Fgf9* expression in cervical loop epithelium becomes labially restricted later than *Activin* and *Fgf3* do in the adjacent labial mesenchyme (Figures 4A and S3; [16]). Therefore, FGF9 alone cannot account for the preferential expression of either gene in labial mesenchyme. *Activin* is so far the earliest gene showing asymmetric expression in incisor dental mesenchyme.

**Figure 7:**
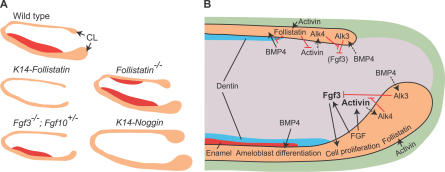
Different Incisor Phenotypes of Several Mouse Mutants and the Signaling Network in the Stem Cell Niche (A) Summary of the sizes of stem cell niches (cervical loops [CL]) and of the distribution of enamel (red) and dental epithelium (orange) in mouse incisors with the indicated genotypes. (B) Model showing the gene regulatory network that controls epithelial stem cell proliferation in the incisor stem cell niche. See text for details.

Collectively, these studies integrate FGF, BMP, Activin, and Follistatin into a signaling network whose balance regulates the proliferation of epithelial stem cells and TA cells in the continuously growing rodent incisor (Figure 7A and 7B). In our model, mesenchymally expressed FGF3 and FGF10 are key effectors of epithelial cell proliferation. BMP4, which is symmetrically expressed in both labial and lingual dental mesenchyme, actively represses *Fgf3* expression, and therefore acts as an endogenous brake on epithelial stem cell and TA cell proliferation. As discussed above, this effect of BMP4 apparently is indirect and involves as yet unidentified signaling events from the epithelium to the mesenchyme regulating *Fgf3* expression. *Fgf10* expression was not affected by BMP. Thus, modulation of FGF-induced epithelial stem cell proliferation by BMP4 and Activin is presumably restricted to the FGF3-dependent component. We hypothesize that the labial cervical loop receives both FGF3 and FGF10 signals and thus becomes hyperplastic relative to the lingual cervical loop, which receives only FGF10. FGF10 may help maintain the basal level of epithelial stem cell proliferation needed to support continuous growth of the tooth and dentinogenesis on the lingual surface [17].

A key feature of the model is that beginning at E15, Activin is expressed asymmetrically. Activin is weakly expressed in lingual dental mesenchyme but strongly expressed in labial dental mesenchyme. On the basis of our findings, we suggest that Activin abrogates the repressive effect of BMP4 on labial *Fgf3* expression and thereby allows FGF3 to promote epithelial stem cell and TA cell proliferation in the labial cervical loop. This would indicate that the balance between two TGF-β superfamily members controls *Fgf3* expression and thereby modulates epithelial stem cell proliferation. The continued intense expression of *Follistatin* in lingual dental epithelium would be expected to antagonize residual Activin in lingual mesenchyme, preserving the repressive effect of BMP on lingual *Fgf3;* whereas on the labial side, there is a gap between *Follistatin*-expressing outer dental epithelium and the subjacent dental papilla mesenchyme. Therefore, Follistatin may not be able to antagonize the activity of Activin, allowing intense expression of *Fgf3* labially. Thus, this regulatory network model explains the increased number of stem cells in the labial compared to the lingual cervical loop, and the asymmetry of the epithelial stem cell niche in the mouse incisor. Although the modulation of proliferation and differentiation in other epithelial stem cell niches has been associated with variations in growth and morphogenesis [1–6], this study is the first to demonstrate how fine tuning of a signaling network within a stem cell niche may directly account for asymmetric organogenesis.

The composition of this gene regulatory network has important implications for organ regeneration. The observation that *Activin, Bmp2, Bmp4, Bmp7, Fgf3,* and *Fgf10* expression is confined to the mesenchyme underlines the importance of the dental papilla mesenchyme in regulation of the epithelial stem cell niche. Significantly, mesenchyme also has a key inductive role in maintaining the regenerative potential of other organs. For example, in hair regeneration, several of these signals were also among the dermal papilla mesenchyme signature genes identified in a recent microarray analysis [18]. The delineation of this network also has evolutionary implications. Although continuous tooth development is often regarded as intrinsic to the rodent incisor, other rodents such as the vole (Microtus sp.) possess continuously growing molars with comparable stem cell niches [10]. Moreover, the Madagascar lemur Aye-aye, *Daubentonia madagascariensis,* a primate, also possesses continuously growing incisors [19]. These observations suggest that the dental epithelial stem cell niche is both robust enough to be retained throughout evolution, yet flexible enough to account for differences in the sizes, shapes, growth rates, and regenerative capacities of teeth in different animals. The fine-tuning of stem cell proliferation by precise modulation of signaling networks may be a general mechanism that accounts for the different regenerative capacities of other organs as well.

## Materials and Methods

### Animals and tissue preparation.

*Follistatin^−/−^* mice on a C57BL/6/J/129S6/SvEv background and *K14-Follistatin* transgenic mice in a B6D2F1 × C57BL/6 mixed background were generated and genotyped as described [8,11,20]. *Fgf3^−/−^* mice, *Fgf10^+/−^* mice, and *K14-Noggin* transgenic mice have been described [13,21,22]. The day of vaginal plug appearance was taken as E0. Staged embryonic heads were fixed in 4% paraformaldehyde (PFA), embedded in paraffin, and serially sectioned at 7 μm. Postnatal mouse heads were decalcified, using 12.5% EDTA containing 2.5% PFA, for 10–14 d and embedded in paraffin. Undecalcified mandibles were dehydrated in a graded ethanol series and embedded in methylmethacrylate [8]. Ground sections (100 μm) were cut sagittally from mandibular incisors.

### In situ hybridization.

[^35^S]-UTP (Amersham, http://www.amersham.com) labeled radioactive in situ hybridization on paraffin sections were performed as described [8]. The following in situ probes were used: murine *Activin βA* [8], *Bmp2, Bmp4, Bmp7* [23], *Follistatin* [20], *Fgf3* [1,16], *Alk3* [24], *Alk4* [25], and rat *Fgf10* [1]. Digitalized images were processed with Adobe Photoshop (http://www.adobe.com). Dark-field images were inverted, false colored with red, and combined with bright-field images [23].

### Tissue culture and bead implantation assays.

Incisor tooth germs from staged embryos were dissected and cultured on 0.1-μm pore size Nuclepore filters at 37 °C at the medium–gas interface using a Trowell-type culture system [26]. Bead implantation assays were performed as described previously [27]. Affi-Gel agarose beads (BioRad, http://www.bio-rad.com) were incubated in Activin A (100 ng/μl), BMP4 (100 ng/μl), FGF3 (50 ng/μl), FGF9 (50 ng/μl), and/or Noggin (500 ng/μl), all from R&D Systems (http://www.rndsystems.com). Control beads were incubated in BSA (Sigma, http://www.sigmaaldrich.com). Beads were soaked in recombinant proteins at 37 °C for 45 min and placed on top of explants or carefully inserted into the incisor explants using fine forceps. After culturing in vitro for 20–24 h, explants were fixed with 100% ice-cold methanol for 2 min, 4% PFA overnight, and processed for whole-mount RNA in situ hybridization as described previously [8]. Some explants were post-fixed in 4% PFA, embedded in gelatin, and sectioned at 50 μm on a vibratome (Microm HM650 V; Microm, http://www.microm-online.com). Dissection and culture of the proximal part of P1 incisor explants was performed as described [1]. Activin A (1.25 ng/μl; kindly provided by M. Hyvönen; [28]) and selective and potent inhibitor of Activin receptor-like kinase (ALK) receptors (SB431542, 0.66 ng/μl; Sigma-Aldrich) were added to the culture medium and, after culture, the explants were fixed in 4% PFA, embedded in paraffin, and serially sectioned.

### Cell proliferation assays.

E18.5 pregnant mice were injected intraperitoneally with 1.5 ml/100 g body weight of BrdU solution (Zymed Laboratories/Invitrogen, http://www.invitrogen.com/antibodies) and killed after 2 h. In in vitro experiments, 10 μM BrdU was added to culture medium for the last 2 h of culture. Embryonic heads and cultured tissues were fixed in 4% PFA, embedded in paraffin, and immunodetected using a BrdU detection kit (Zymed Laboratories/Invitrogen). The cell proliferation indices were determined by counting the BrdU-positive and -negative cells in the cervical loop epithelium and mesenchyme in defined areas. The cells of the labial- and lingual-side mesenchyme were determined from a 200 μm × 400 μm–wide region parallel to epithelium. The initial secretory-stage odontoblasts were chosen for the border of counting cells in the labial and lingual dental epithelium, respectively. The mean values were calculated from the two independent observers' data pools. When the numbers of proliferating cells in *K14-Follistatin* and *Follistatin^−/−^* were compared to the wild type (Student *t*-test), all the differences were highly significant.

## Supporting Information

Figure S1 Analyses of Epithelial and Mesenchymal Cell Proliferation in Incisor (A and B) *Follistatin* is strongly expressed in the epithelium of *K14-Follistatin* newborn mouse incisor.
(C) The BrdU index (the relative amount of BrdU-positive cells from the total amount of cells) showed significant differences both in the epithelium and mesenchyme between the wild-type, *K14-Follistatin,* and *Follistatin^−/−^* mice in the cervical loop area of the incisors.
(D–F) E15 incisor explants were cultured in vitro for 24 h with beads containing the indicated recombinant proteins, and cell proliferation analyzed by BrdU incorporation. (D) FGF3 beads stimulated marked cell proliferation in adjacent dental epithelium. (E) Activin A beads and (F) BSA control beads had no effect.
Scale bar represents 200 μm (A and B).
(1.0 MB PDF)

Figure S2 In Situ Hybridization Analysis of *Bmp2* and *Bmp7* Expression in the Developing Incisor from E14–16 *Bmp2* and *Bmp7* are expressed symmetrically in incisor mesenchyme. *Bmp7* is also expressed on the labial side in differentiating ameloblasts. Scale bars represent 200 μm.
(3.0 MB PDF)

Figure S3 Ectopic *Fgf3* Expression in *K14-Noggin* Incisor Mesenchyme and Expression of *Fgf9* in Cervical Loop Epithelium (A) In *K14-Noggin* mouse incisors at the newborn stage, ectopic *Fgf3* is detected in lingual dental mesenchyme directly underneath lingual dental epithelium.
(B) At E17, *Fgf9* is expressed in both labial and lingual dental epithelium (arrows).
(C) At P2, *Fgf9* expression is confined only to labial-side dental epithelium (arrow; [14]).
Scale bars represent 200 μm.
(504 KB PDF)

## Abbreviations

Alk: activin receptor-like kinase
BMP: bone morphogenic protein
BrdU: bromodeoxyuridine
E: embryonic day
FGF: fibroblast growth factor
K14: keratin 14
P: postnatal day
TA: transit amplifying
TGF-β: transforming growth factor β
