# Supplementary material for: An Integrated Gene Regulatory Network Controls Stem Cell Proliferation in Teeth
Source: PLoS Biol. 2007 Jun 12;5(6):e159. doi: 10.1371/journal.pbio.0050159 (PMC1885832; doi:10.1371/journal.pbio.0050159)
Supplement: Supplementary file 1 [file pbio.0050159.sg001.pdf]

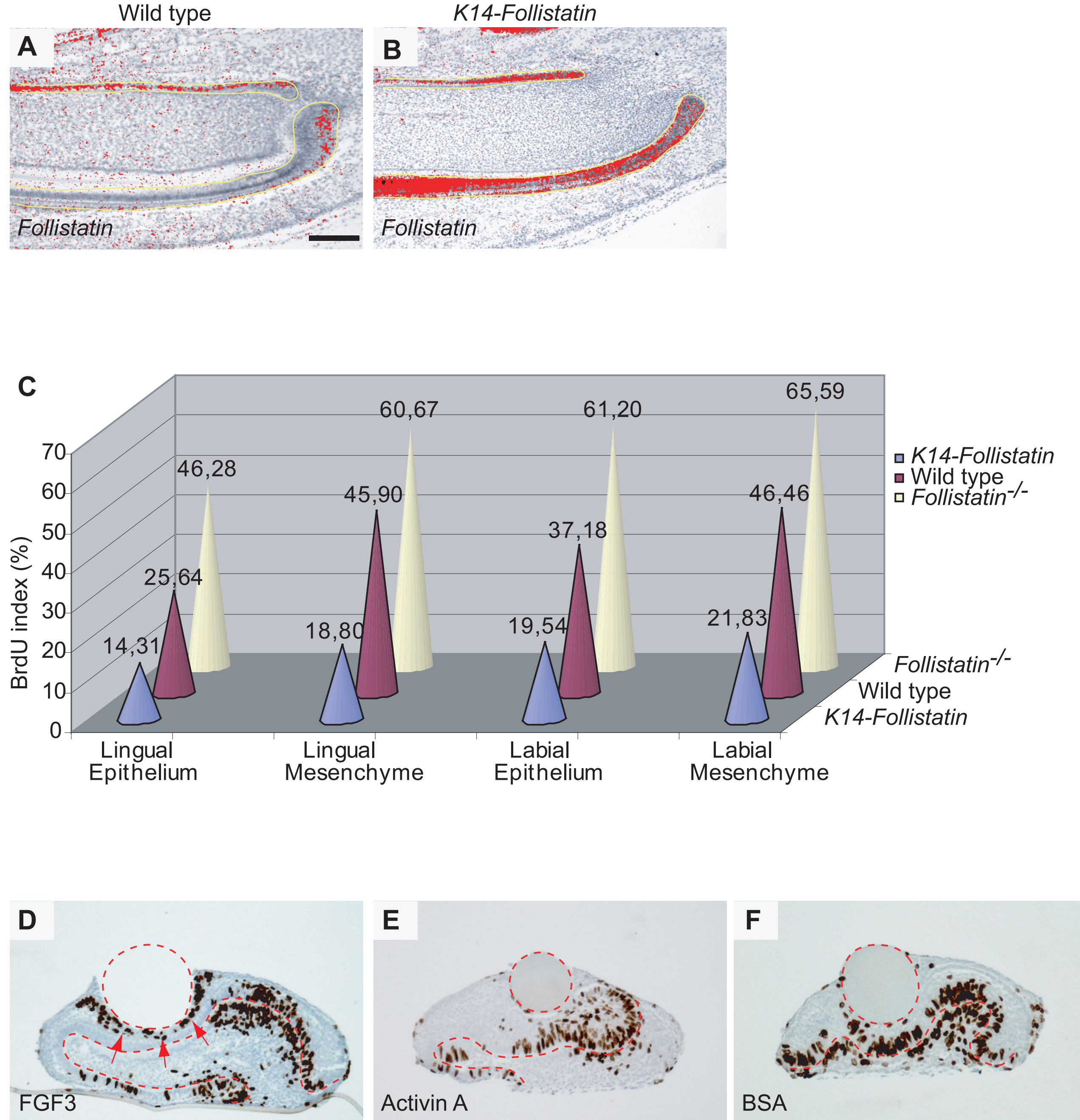

Wild type
K14-Follistatin
A
B
Follistatin
Follistatin
C
70
60
50
40
30
20
10
0
BrdU index (%)
14,31
25,64
46,28
18,80
45,90
60,67
19,54
37,18
61,20
21,83
46,46
65,59
K14-Follistatin
Wild type
Follistatin-/-
Follistatin-/-
Wild type
K14-Follistatin
Lingual Epithelium
Lingual Mesenchyme
Labial Epithelium
Labial Mesenchyme
D
E
F
FGF3
Activin A
BSA


**FIGURE S1**
