# Supplementary material for: An Integrated Gene Regulatory Network Controls Stem Cell Proliferation in Teeth
Source: PLoS Biol. 2007 Jun 12;5(6):e159. doi: 10.1371/journal.pbio.0050159 (PMC1885832; doi:10.1371/journal.pbio.0050159)
Supplement: Supplementary file 2 [file pbio.0050159.sg002.pdf]

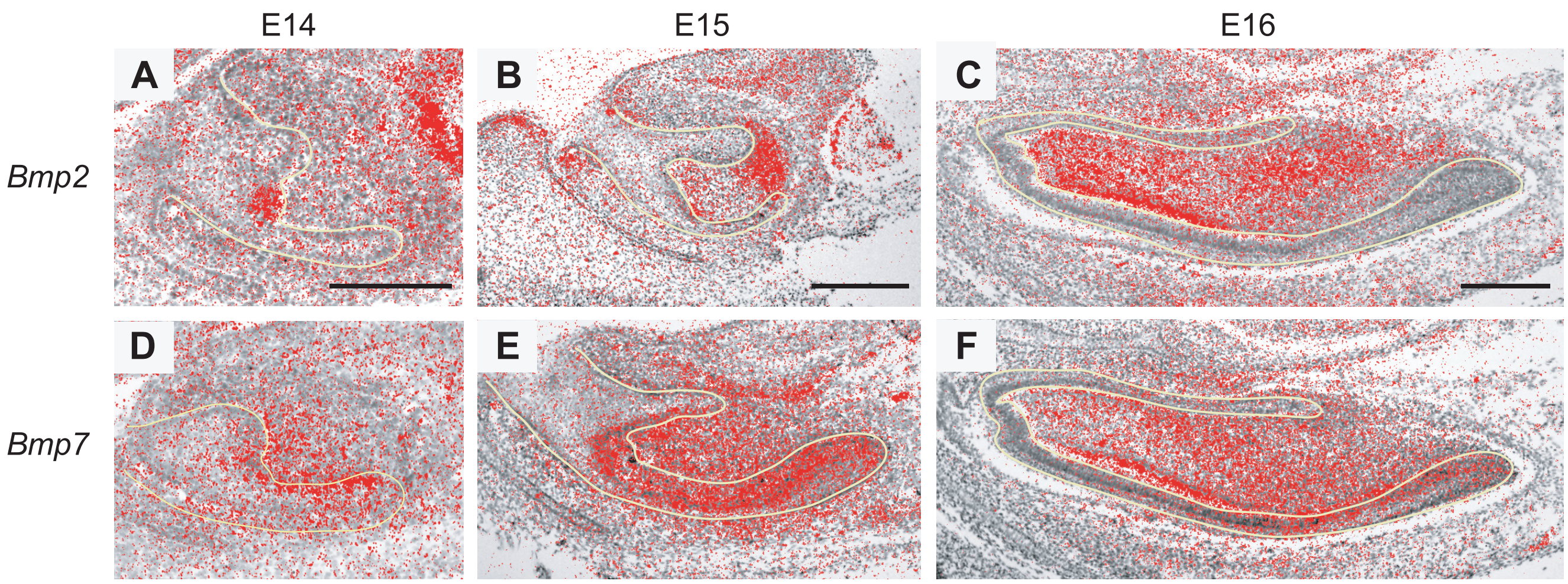

FIGURE S2
